# An implementation study of the crisis resolution team model in Norway: Are the crisis resolution teams fulfilling their role?

**DOI:** 10.1186/1472-6963-11-96

**Published:** 2011-05-10

**Authors:** Nina Hasselberg, Rolf W Gråwe, Sonia Johnson, Torleif Ruud

**Affiliations:** 1 R&D Department, Mental Health Services, Akershus University Hospital and Institute of Clinical Medicine, University Oslo, Norway; 2 Department of Research and Development, Alcohol and Drug Treatment Health Trust in Central Norway; Norwegian Centre for Addiction Research, University of Oslo, Norway; 3 Department of Mental Health Sciences, University College London, London, UK; 4 R&D Department, Mental Health Services, Akershus University Hospital and Professor, Institute of Clinical Medicine, University Oslo, Norway

**Keywords:** acute psychiatric services, crisis resolution teams, mental health services, implementation study, patient characteristics

## Abstract

**Background:**

The establishment of crisis resolution teams (CRTs) is part of the national mental health policy in several Western countries. The purpose of the present study is to describe characteristics of CRTs and their patients, explore the differences between CRTs, and examine whether the CRTs in Norway are organized according to the international CRT model.

**Methods:**

The study was a naturalistic study of eight CRTs and 680 patients referred to these teams in Norway. Mental health problems were assessed using the Health of the Nation Outcome Scales (HoNOS), Global Assessment of Functioning Scales (GAF) and the International Statistical Classification of Diseases and Related Health Problems, 10^th ^Revision (ICD-10).

**Results:**

None of the CRTs operated 24 hours a day, seven days a week (24/7 availability) or had gate-keeping functions for acute wards. The CRTs also treated patients who were not considered for hospital admission. Forty per cent of patients waited more than 24 hours for treatment. Fourteen per cent had psychotic symptoms, and 69% had affective symptoms. There were significant variations between teams in patients' total severity of symptoms and social problems, but no variations between teams with respect to patients' aggressive behaviour, non-accidental self-injury, substance abuse or psychotic symptoms. There was a tendency for teams operating extended hours to treat patients with more severe mental illnesses.

**Conclusions:**

The CRT model has been implemented in Norway without a rapid response, gate-keeping function and 24/7 availability. These findings indicate that the CRTs do not completely fulfil their intended role in the mental health system.

## Background

The key characteristics of CRT model are separate multidisciplinary mobile teams offering rapid short term emergency services in the community, as an alternative to inpatient admission [1]. CRTs are intended to operate 24 hours, 7 days per week with a gate keeping function to acute wards. The target group is patients with psychosis or other mental health problems so severe and acute that without the involvement of a CRT, acute admission would usually be necessary [1-5]. Establishing CRTs is a part of the national mental health policy in several countries. In the UK, CRTs have been rapidly implemented across the country with 343 teams in place in 2006/07 [6], and in Norway 35 of the 75 community mental health centres (CMHCs) had established a CRT by 2008 [7]. Both CRTs and assertive outreach teams are intended to manage episodes of acute mental illness without admitting the patient to hospital. Assertive outreach teams provide intensive long-term community-based support for frequently relapsing and difficult-to-engage patients enrolled in their programme, while CRTs provide crisis resolution to anyone considered to be in the target group [8,9].

A Cochrane review of randomized controlled studies on home care crisis treatment from the 1960s to the 1980s showed that such treatment could be effective [10], but this may not be representative of the recent model for CRTs. A later review found that CRTs were promising, but could not draw conclusions because of limited research [11]. In the last decade, only one randomized controlled trial of a CRT has been completed [12]. Johnson et al found a reduction in hospital admissions and a small increase in the satisfaction of patients receiving CRT care compared with standard care. Other uncontrolled recent studies also suggest that the introduction of CRTs was associated with a reduction in admissions [9,13-16] and there is some evidence that service users are more satisfied with CRTs than with standard care [13,17-19].

Some recent studies have described characteristics of CRTs. Glover et al used routine data to analyse national changes following the implementation of the CRT model across the UK [9]. They found that teams operating 24 hours a day, seven days a week (24/7 availability) were most likely to be associated with reduced admissions. Onyett et al examined how the CRT model has been implemented in the UK and identified 243 teams [20]. Almost all teams included nurses, the majority of teams included support workers, and just under half included psychiatrists. Other professions were not well represented. Sixty-eight per cent reported that they were gate-keepers to acute wards and 54% offered 24/7 availability.

Regarding patient characteristics of the CRTs, in the randomized controlled trial of Johnson et al, patients' average age was 38 years, about half were men, half were living alone, half were from ethnic minorities, most were unemployed, and 37% had a psychotic disorder [12]. In a non-randomized study by Johnson et al, patients' characteristics were similar except for some minor differences in the number of ethnic minorities, unemployment, and patients with psychotic diagnoses [13]. In a Norwegian study of one CRT, 27% had hallucinations or delusions [21]. In studies of home-care acute psychiatric treatment, based on data collected before the government proposed the establishment of nationwide CRTs in the UK [2], it was found that 53-62% of patients had psychotic disorders [22-25].

Several authors have pointed out that there is a gap between models based on what is known about effective treatment, and the implementation of effective routine clinical practice [26-28]. Tansella and Thornicroft [26] described three phases, including different barriers or facilitators at the national, local and individual levels, in understanding the translation of knowledge in the health science into routine clinical practice. The three phases are called adoption in principle, early implementation and persistence of implementation. This is a study of how the transfer of knowledge from the CRT model has been implemented into routine clinical practice in Norway.

The aims of the present study were to a) describe the characteristics of Norwegian CRTs and their patients, b) examine if there are differences between the CRTs with reference to key team characteristics and patients' mental health problems, c) examine if the teams cluster into particular groups with shared characteristics, and d) examine whether the CRTs in Norway are organized according to the international CRT model.

## Methods

### Study design

The study was a naturalistic study on eight CRTs and their patients in Norway, as part of the Multicentre Study on Acute Psychiatry (MAP) in Norway. The multicentre study was planned and implemented by a national network for the evaluation of acute psychiatric services.

### Setting

In 2005, the Norwegian health authorities decided to implement the CRT model in Norway, inspired by the implementation of CRTs in the UK. The implementation of CRTs in Norway was proposed to increase accessibility to specialized mental health services for patients experiencing acute mental health crisis. The teams were to offer rapid assessment and 24/7 availability, and be an alternative treatment to acute admission.

Norway has 4.8 million inhabitants. There are large areas with low population density which implies longer distances to acute wards for the patients and longer distances to patients' residences for the staff of the mental health services. The national mental health system for adults consists of three service levels: at the first level there are GPs and mental health teams in primary care settings run by the 430 municipalities. Some municipalities have residential or sheltered accommodation. At the second level, there are 75 CMHCs. The CMHCs comprise different types of care units and teams. The outpatient teams comprise general outpatient teams, psychosis/rehabilitation/ambulatory teams, drug/alcohol teams and day/group teams. Some teams provide inpatient treatment at the CMHCs [7]. Assertive outreach teams are in the early stages of implementation in Norway. At the third level, there are psychiatric hospital wards, including acute wards (21 beds per 100,000 inhabitants).

### Sample

The sample consisted of all 680 patients seen by eight CRTs in 2005 or the beginning of 2006. All patients 18 years or older, who had face-to-face consultations with the CRT, were included in the study. The inclusion period was three months, but could be prolonged to include 60 patients from each team. The number of 60 patients was chosen to include a reasonable sample of patients from each team for comparative data analysis and to give a picture of the implementation process of the CRT model. The number of patients included by each team ranged from 46-147. There were no exclusion criteria.

All the CRTs in Norway at that time took part in the study, except one that had recently carried out a study of its own [21]. The CRTs were from all parts of the country. Two teams were in urban areas, and the other six were in smaller towns or more rural areas. None of the catchment areas of the CRTs can be characterized as highly deprived. One of the teams is situated within an area with a significant number of people from minority ethnic groups (26%, compared to 2-16% for the other CRTs). The eight CRTs in this study covered 15.4% of the total population in Norway.

All patients were included. Written consent was not requested as the Regional Committee on Ethics in Medical Research agreed that, for ethical reasons, it was important to include all patients in need of acute treatment, especially those with severe mental illness who probably would not have given written consent.

### Registration form, instruments and data collection

A registration form was used to collect data from each treatment episode. The form was developed in the network doing the multicentre study, and the final version was based on experiences of earlier pilot drafts. The data on patients included socio-demographic and clinical data. Type and severity of psychiatric problems and level of functioning were assessed using the International Statistical Classification of Diseases and Related Health Problems, 10^th ^revision (ICD-10) for diagnoses [29], the Health of the Nation Outcome Scale (HoNOS) [30] and Global Assessment of Function Scales (GAF) [31-33]. The HoNOS has 12 items with a five-point scale (from 0-4) regarding severity of clinical and social problems. We used a split version of the GAF consisting of two scales ranging from 1-100 for symptom severity and functional impairment, respectively. Staff members who participated in the study received half a day of training in the use of the HoNOS, as provided in the UK, and all clinicians were trained in using the GAF as it was the routine measure required for all treatment episodes in the mental health services in Norway. Other studies with the same training of clinicians have repeatedly shown acceptable inter-rater reliability (intra-class correlation coefficient of. 60-.89) for the HoNOS subscales, with the exception of scale 8 [34]. These reliability data were from the approved Norwegian translation of HoNOS as used in this study. Scale 8 was therefore excluded from the analysis of single HoNOS scales. Studies have indicated moderately high internal consistency and low item redundancy of the HoNOS sum score, and therefore support the use of sum scores as a meaningful summary of severity of symptoms [35]. The Alcohol Use Scale (AUS) and the Drug Use Scale (DUS) were also used at admission [36,37] to rate the severity of alcohol and drug use, respectively. These are five-point scales ranging from "abstinence" (1) to "abuse" (3) to "addiction with hospitalization" (5). A suicidal behaviour scale (suicidal ideation, plans or attempts) administered at the time of referral was designed in collaboration with the National Centre for Prevention of Suicide [38]. ICD-10 diagnoses were made during the treatment.

Staff members from each team filled in the registration forms on the patients they were treating, and one team member coordinated the data collection for the team.

A questionnaire on how the CRT was organized and operated was completed by the leader of each team. This included information on catchment area, opening hours, number of team members and their profession, accessibility to beds, and availability of psychosis teams (early intervention teams and/or case management teams) or community mental health teams in the catchment area.

### Approval from authorities and contributions from user groups

The study was approved by the Regional Ethical Committee for Research in Health and by The Norwegian Data Inspectorate. The Directorate of Health and Social Affairs gave consent for the use of information from the health services.

Representatives of the user organizations Mental Health Norway and The National Association of Relatives in Mental Health participated in a reference group and in the workshops for planning and preparation of the study.

### Data analysis

For HoNOS scales with missing data (5.5% across scales) the rating was set to 0. This was considered to be the most probable rating based on the skewed distribution, with most patients rated 0, and it was assumed that clinicians would forget to mark the rating when there was no indication of problems. In addition, this was chosen in favour of imputation because it was the most conservative way to measure the patients' severity of mental health problems.

Diagnoses were missing for 54% of patients in one team, 17% in another team, and 3-10% for the other teams. In Norway, only physicians and psychologists are authorized to make ICD-10 diagnoses. The teams with the most missing values on the diagnosis variable operated without a physicians/psychiatrist or psychologist as a part of the team and with nurses and social workers as the majority of staff. In these teams, diagnoses were made by physicians who were not a part of the team. These physicians took part in some consultations that focused on issues such as psycho pharmacological treatment, admissions to acute psychiatric ward, suicidal risk, violence risk or compulsory admission. In addition, the staff did use previous diagnoses made by physicians/psychologists in other mental health services.

For this reason, HoNOS scales were used instead of diagnosis in the analyses of type and severity of psychiatric problems. There was no significant difference between patients with or without diagnosis on the sum scale of HoNOS score or GAF symptoms and functioning score.

Continuous and some ordinal variables are reported with means and standard deviations and categorical and some ordinal variables are reported with frequencies and percentages. Independent sample *t*-tests, chi-squared tests, one-way analysis of variance (ANOVA), Mann-Whitney *U *and Kruskal-Wallis tests were used to test for statistically significant differences. For skewed variables, both parametric and non-parametric tests were used and results did not differ. Multiple comparisons with Bonferroni post hoc corrections were used to reduce the probability of type 1 errors. Thus, with an alpha level of 0.05, the individual error rate was reduced to 0.002 when using 25 items (0.05 divided by 25).

Hierarchical cluster analysis was used to identify homogeneous groups of CRTs based on key team characteristics. A dissimilarity matrix was calculated using squared Euclidean distance and clustering performed by Ward's method. Comparison of identified clusters was made by chi-square, *t*-tests and Mann-Whitney *U *tests on key patient characteristics. Both SPSS and Clustan showed the same results.

SPSS software (version 15 for Windows; SPSS Inc., Chicago) was used for data analysis. A significance level of 0.05 was used.

## Results

### Characteristics of the CRTs

Table 1 shows some characteristics of the organization and policy of the eight CRTs in this study.

**Table 1:** Characteristics of CRTs (n = 8)

Population of catchment areas: mean (range)	87,000 (65,000-115,000)
	
Opening hours: n	
24/7 (24 hours a day, 7 days a week)	None
Availability at night by phone	2
Team operates extended hours and at weekends	3
Team operates extended hours	1
Team operates office hours 5 days a week	4
Gate-keeping of admissions to acute psychiatric wards: n (%)	0
Staffing: mean (range)	
Number of team members (FTE)	9.1 (4.3-19.2)
Number of clinical disciplines	3.5 (3-5)
Psychiatrist/phyicians: mean (range)	0.6 (0-1.4)
Psychologist/specialist in psychology	1.5 (0-2.5)
Nurse/psychiatric nurse	6.7 (1.5-6.2)
Social workers	0.9 (0-1)
Other disciplines	1.8 (0-7.3)
Office staff	0.6 (0-2)
Team with a full-time psychiatrist: n (%)	3 (37.5)
Other characteristics: n if not otherwise specified	
Accepting patients for consultation without referral	7
Accessibility to beds (not acute inpatient beds)	2
Authority to admit patients to acute in-patient wards	4
How fast do the teams respond to referrals: range in hours	12-48
Psychosis team in the area	5

None of the teams had 24/7 availability or gate-keeping functions to acute inpatient wards, and they all treated some who were not considered for hospital admission.

The number of team members varied substantially (range = 4-19 full time equivalent staff members, which was 0.5-2.0 staff members per 10,000 inhabitants). Some of the CRTs had been established recently, while others had been in operation for longer periods of time (range = 0-6 years). The CRTs were led by different professionals (psychiatrist, psychologist or psychiatric nurse). The CRTs used a team approach to patients, and two clinicians usually participated in each consultation.

The mean waiting time for admission to the CRTs was 1.6 days (*SD *= 10.4), and the median waiting time was one day. Approximately 40% of patients waited more than 24 hours. Patients with psychotic symptoms waited significantly less time than patients with other mental health problems.

### Patient characteristics

The socio-demographic and clinical characteristics of the 680 patients are presented in Table 2.

**Table 2:** Characteristics of patients (n = 680), and variations between CRTs (n = 8)

Variables	Total sample	Significance of differences between teams*
Socio-demographic variables		
Age (years), mean (*SD*)	40.1 (15.1)	0.066
Gender: n (%) female	396 (58.8)	0.507
Single, divorced or widowed, n (%) ͣ	415 (62.5)	0.022**
Living alone, n (%)	396 (58.2)	<0.001**
Employed at present, n (%)	175 (25.7)	0.006**
Not receiving benefit or disablement pension, n (%) ͣ	258 (38.0)	0.035**
Clinical variables		
Clinical diagnosis (ICD 10) n (%)		
F 10-19 Substance use disorders	53 (7.8)	0.008**
F 20-29: Schizophrenia spectrum disorders	60 (8.8)	0.002**
F 30-39: Mood/affective disorders	220 (32.4)	0.003**
F 40-49: Neurotic, stress-related and somatoform disorders	147 (21.6)	<0.001**
F 60-69: Disorders of adult personality and behaviour	30 (4.4)	<0.001**
Missing diagnosis	119 (17.5)	0.001**
GAF: mean (*SD*)		
Symptoms:	48.4 (11.6)	<0.001**
Functioning:	49.6 (12.6)	<0.001**
Substance abuse or dependency: n (%)		
Alcohol (AUS):	74 (10.9)	0.001**
Drugs (DUS):	73 (10.7)	0.100
Suicidality: n (%)		
No suicidal thoughts/plans	260 (39.8)	<0.001**
Passive death wishes/suicidal thoughts, no concrete plans	261 (39.9)	
Concrete suicidal plans/self-injury, but no death intentions	110 (16.8)	
Self-injury/death intentions	23 (3.5)	
Severity of clinical and social problems: Mean (*SD*)		
HoNOS Total score	12.5 (6.26)	< 0.001**
HoNOS Total symptom severity (HoNOS 1-8):	7.6 (3.72)	< 0.001**
HoNOS Total social problem severity (HoNOS 9-12):	5.0 (3.53)	< 0.001**
HoNOS items: n (%) (score 2-4 on scale 0-4)		
HoNOS 1 Overactive, aggressive or disruptive behaviour	115 (16.9)	0.442
HoNOS 2 Non-accidental self-injury	126 (18.5)	0.447
HoNOS 3 Problems with drinking or drug-taking	130 (19.1)	0.074
HoNOS 6 Problems with hallucinations and delusions	99 (14.1)	0.273
HoNOS 7 Problems with depressed mood	467 (68.7)	0.001**
HoNOS 9 Problems in relationships	322 (47.4)	0.001**
Other characteristics		
Previous contact with the mental health service: n (%)	401 (59.0)	<0.001**
Emergency referrals: n (%)	489 (71.9)	<0.001**
Self-referrals: n (%)	172 (25.3)	<0.001**
Pharmacological treatment: n (%)	241 (35.4)	< 0.001**
Waiting time: mean (*SD*)	1.6 (10.4)	0.137

*p values from chi-square tests, ANOVA and Kruskal-Wallis tests **significant differences between teams

ͣ not significant using Bonferroni adjustment for multiple comparisons

Most patients were aged between 20 and 50 years, slightly more than half were women. Approximately half of the patients were unmarried and living alone, and one-quarter were in paid employment. Twenty-three patients (2%) were homeless. Twelve patients (1%) were not of Norwegian ethnicity, compared to 8% of the general population of Norway. The majority of patients had primarily mood and anxiety disorders, and 14% had psychotic symptoms.

About 60% of the total sample had had previous contact with the mental health services. In the past 12 months, 38% of patients had received treatment at an outpatient clinic, and 22% had been in an inpatient ward. Patients with previous contact with the mental health services had significantly more severe mental health problems on most clinical measures (HoNOS total score; 13.6/11.0, *p *<.001, GAF symptoms; 46.7/50.7, *p *<. 001, and GAF functioning; 47.5/52.1, p <. 001).

Three in four were emergency referrals and 25% had self-referred to the CRT. Patients with emergency referrals had significantly more severe mental health problems on most clinical measures than those who were not referred as an emergency (HoNOS total score; 13.2/10.7, *p *<. 001, GAF symptoms; 47.2/51.2, *p *<. 001, and GAF functioning; 48.5/52.3, *p *<. 001). Those who self-referred did not differ significantly on any clinical measures from those who were referred (HoNOS total score; 12.9/12.4, *p *= 0.42, GAF symptoms; 47.5/48.6, *p *= 0.28., and GAF functioning; 48.0/50.1, *p *= 0.05).

### Variation between teams and their patients

Table 2 shows comparisons between the CRTs on patients' socio-demographic and clinical characteristics. After Bonferroni adjustment for multiple comparisons, there were no significant differences between the CRTs on socio-demographic variables, except whether patients were living alone, and whether patients were employed, with the two urban CRTs serving more unemployed patients. There were no significant differences between the CRTs on proportions of patients with overactive, aggressive or disruptive behaviour (HoNOS 1), non-accidental self-injury (HoNOS 2), problems with drinking or drug-taking (HoNOS 3), or problems with hallucinations and delusions (HoNOS 6). There were significant differences between teams on most other clinical variables.

The hierarchical cluster analysis indicated that the CRTs clustered on operating during office hours (except for one team) and lack of a psychosis team in the catchment area. However, the cluster analysis did not give a consistent clustering on the combination on these variables and the degree to which the teams operated with a full-time psychiatrist. The most distinct cluster identified by the analysis was the clustering on teams that operated during office hours (except for one team), which made a separate cluster in both the 2- and 3-cluster structure. Due to this, we chose to analyse differences between teams in respect of whether the teams operated during office hours and whether there was a psychosis team in the area.

As shown in Table 3, after Bonferroni adjustment for multiple comparisons, patients admitted during CRTs operating office hours were significantly less likely to live alone and had lower scores on all HoNOS total scales. In the CRTs that operated with no psychosis team in their catchment area, the patients were significantly less likely to have problems of suicidality and had higher GAF functioning scores.

**Table 3:** Variation between groups of teams based on opening hours and psychosis team in the area

Variables	Opening hours			Psychosis team in the area		
	Extended	Not extended	*p *value*	Yes	No	*p *value*
Teams	n = 4	n = 4		n = 5	n = 3	
Patients	n = 406	n = 274		n = 427	n = 253	
Living alone n (%)	264 (65.0)	132 (48.2)	<0.001**	242 (56.7)	154 (60.9)	0.284
Employed n (%)	99 (24.4)	76 (27.7)	0.327	105 (24.6)	70 (27.7)	0.375
GAF symptoms, mean (*SD*)	47.8 (10.6)	49.2 (12.2)	0.140	48.0 (11.1)	49.0 (12.5)	0.238
GAF functioning, mean (*SD*)	47.2 (11.8)	50.4 (12.8)	0.156	48.5 (11.6)	51.3 (13.8)	0.005**
Suicidal thoughts/plans n (%)	244 (63.7)	150 (55.4)	0.031 ͣ	269 (64.7)	125 (52.5)	0.002**
HoNOS Total, mean (*SD*)	13.4 (6.5)	11.2 (5.7)	<0.001 **	12.3 (6.2)	12.9 (6.4)	0.219
HoNOS Total symptom severity mean (*SD*)	7.9 (3.9)	7.1 (3.4)	0.004**	7.4 (3.7)	7.8 (3.7)	0.128
HoNOS Total social problem severity, mean (*SD*)	5.5 (3.6)	4.2 (3.2)	<0.001**	4.9 (3.4)	5.1 (3.8)	0.563
Self-referrals n (%)	116 (28.6)	56 (20.3)	0.017 ͣ	101 (23.7)	71 (30.3)	0.201
Waiting time days, mean (*SD*)	1.9 (12.9)	0.9 (2.4)	0.039 ͣ	1.3 (4.4)	1.9 (15.7)	0.441

*p values are from chi-square tests for categorical variables, *t*-tests for continuous variables and Mann-Whitney *U *tests for ordinal variables

**significant differences between CRTs

ͣ not significant using Bonferroni adjustment for multiple comparisons

## Discussion

The purpose of this study was to describe the characteristics of Norwegian CRTs and their patients, examine any differences and/or clusters between the CRTs, and examine whether the CRTs in Norway are organized according to the international CRT model.

### Characteristics of the teams

We found that, in Norway, the CRTs did not have 24/7 availability or gate-keeping functions for acute wards, average waiting time was about one and a half days, and the CRTs appeared to treat some patients who were not considered for hospital admission.

Operating without a gate-keeping function and 24/7 availability, with a waiting time of approximately one and a half days and not focusing on patients for whom hospital admission is considered, are fundamental departures from the CRT model. Fidelity scales of assessment of critical ingredients have not been established for CRTs as they have for assertive outreach teams. However, there is some consensus on the key characteristics of the CRT model [1,2,5]. These are a separate multidisciplinary team, the capability to deliver a full range of emergency psychiatric interventions in the community, targeting severe emergencies in which inpatient admission would otherwise be required, 24/7 availability, a psychiatrist working within the team, rapid emergency assessments, response within one hour if required, and gate-keeping functions to acute wards.

There are a number of possible explanations for not having implemented the whole CRT model in Norway. Firstly, it is less expensive to operate without 24/7 availability. Secondly, the geography of Norway, which is characterized by low density of population compared to the UK, makes rapid response and home treatment more challenging. Thirdly, a gate-keeping function to acute wards requires a transfer of authority from acute wards to CRTs. This has not been done in Norway. Fourthly, the practice of treating patients who are not considered for hospital admission may be explained by a need to reduce pressure on other mental health services. It is therefore possible that the CRTs are reducing emergency referrals to outpatient clinics more than to the acute wards. Fifthly, independent of national and international guidelines, local and national variations in resources and available clinical staff can affect team composition at times. In addition, there is a greater risk of local variation when the national guidelines for CRTs in Norway can be criticized for being vague.

Because Norwegian CRTs also treat patients who are not considered for hospital admission, they may have a lower threshold for the initial assessment of patients. This may make it possible for CRTs to pre-empt a full blown crisis by intervening before problems become severe. Evidence is emerging for the importance of early detection and intervention for people who may be developing signs of mental illness [39], though the aim of the CRTs is to give emergency community treatment to patients already in severe acute crises.

### Patient characteristics

Our study showed that 14% of patients admitted by the CRTs had psychotic symptoms, one-quarter of the patients in our study were employed and three in four were emergency referrals. In both the UK and Norway, CRT services are intended to target patients with psychosis and other severe mental health problems [2,3]. The smaller proportion of patients with psychotic symptoms in Norway cannot be explained by differences in the size of the catchment areas in the UK and Norway (63,000 in the Islington area [13] compared with a mean population of 87,000 in the Norwegian CRT catchment areas).

Another possible explanation for the low proportion of patients with psychotic symptoms could be that patients with psychosis are treated by psychosis teams at CMHCs, although three of the CRTs did not have a psychosis team in their catchment area and there were no significant differences between CRTs in terms of admission of patients with psychotic symptoms.

A difference between Norway and the UK is that CRTs in Norway accept self-referrals, but in the UK they do not, even though the CRTs in the UK allow direct referrals from former service users and their families or carers [2]. In this study, about one-quarter of patients self-referred to Norwegian CRTs. This may contribute to the CRTs in Norway reaching patients with less acute needs than in the UK, although there were no significant differences between self-referrals and those who were referred by others.

Nevertheless, the above findings on the proportion of patients with psychotic symptoms, persons fully employed and emergency referrals indicate that Norwegian CRTs serve patients with less severe mental illnesses than the CRTs in the UK.

### Variation between teams

The results from this part of the study must be interpreted in light of the fact that there are substantial differences between teams in the way they operate and are organized, and the small number of teams (eight CRTs).

As discussed above, CRTs should serve patients with severe mental symptoms. There were no significant differences between the CRTs on clinically relevant variables regarding the degree to which they treat patients with overactive, aggressive or disruptive behaviour, non-accidental self-injury, problems with drinking or drug-taking, or with psychotic symptoms. This may indicate that there are no significant differences between the CRTs in their admission assessments for these patients.

There was a tendency for teams that operate extended opening hours to treat patients with more severe mental illnesses, but the same consistent pattern of differences did not appear when comparison was made between CRTs operating with a psychosis team in their catchment area and those without. In addition, whether CRTs were operating with a full-time psychiatrist did not seem to make any difference to the severity of psychiatric problems.

Opening hours was the most distinct cluster in our hierarchical cluster analysis. Four of the CRTs in this study operated during office hours only and none of the teams operated with 24/7 availability. This study is not a randomized controlled trial and therefore one cannot draw causality conclusions between the variables measuring severity of mental symptoms and opening hours. There may be other confounding variables. However, patients experience mental health crises in the evenings, at night and on weekends and it is difficult for Norwegian CRTs to operate as an adequate alternative to inpatient treatment if they do not operate during these hours. Local mental health care in these areas provides only casualty clinics and acute inpatient wards outside office hours.

### Implementation

Tansella and Thornicroft [26] described three phases, including different barriers or facilitators at the national, local and individual levels, in understanding the translation of knowledge in the mental health science into routine clinical practice. In the first phase, called adoption in principle, the authors emphasize the importance of setting a policy priority at national level and having clinical guidelines. The establishment of CRTs in Norway was a priority of national mental health authorities, but the teams were not given the resources to operate with 24/7 availability or gate-keeping authority to acute wards. Clinical guidelines were developed in Norway, but these seemed to be less specific than similar guidelines from the UK, giving a greater risk of local variations. On an individual level, the lack of full-time consultant psychiatrists at CRTs in Norway may be related to more general problems in the mental health services, with a limited number of psychiatrists and problems recruiting psychiatrists to vacant positions. This lack of input from consultant psychiatrists makes the CRTs less multidisciplinary, and two teams in our study included mainly nurses and social workers.

While the policy implementation guidelines in the UK look relatively specific, there have been some difficulties in implementing CRTs in the UK too. Onyett et al found 68% of the CRTs in the UK in 2005 reported being gate-keepers to inpatient beds and 54% offered a 24/7 service [20]. A report by the National Audit Office [40] found that the introduction of CRTs had successfully reduced pressure on beds and supported earlier discharge from acute wards, but they found wide regional variations, particularly in the lack of consultant psychiatrists. In addition, they found that of 500 admissions, only half had been assessed by the CRT staff before admission (gate-keeping).

### Strengths and limitations

The main strength of our study is that it is a naturalistic study of nearly all patients treated by the CRTs in Norway at the time of the study. Both the variety within the sample, the size of the sample, the length of the registration period, and the different geographical locations of the CRTs suggest that the data may be considered to be representative of such teams in Norway. Since the data collection, more CRTs have been established (35 of the 75 CMHCs had established a CRT by 2008) in Norway. These 35 CRTs were operating without a gate-keeping function and 24/7 availability and there was still a lack of full-time psychiatrist in these teams. About half of the teams operated extended hours. This indicates that the way the CRTs are organized and operate have not changed significantly since our data-collection and that our data was still representative for these teams [7].

The lack of randomization and control group are important limitations, and causality cannot be shown from this study. The multicentre design meant that many raters participated, which may have introduced some uncontrolled error variances, even though the project coordinators of each team participated in developing the registration forms and were responsible for instructing the other raters and the data collection by their team.

## Conclusion

In our study, we found that the CRTs in Norway did not implement the whole CRT model and this may lead to the result that CRTs were only reaching part of the target group. Norwegian CRTs do not serve as an adequate alternative to admission in the same way as international CRTs and therefore do not completely fulfil their role in the mental health system. For fuller implementation of the CRT model, fidelity scales and supporting toolkits for achieving fidelity might be useful. A further investigation of barriers to implementation is recommended.

## Competing interests

The authors declare that they have no competing interests.

## Authors' contributions

TR, RWG and NH designed the study and formulated the research questions. NH conducted the literature search. NH performed the statistical analysis and interpreted the data with significant support from SJ and TR. The manuscript was written by NH and substantially revised by TR, SJ and RWG. The final version of the manuscript was prepared by and revised by all authors. TR was head supervisor of this manuscript and project leader of the Multicentre Study of Acute Psychiatry in Norway (MAP).

## Pre-publication history

The pre-publication history for this paper can be accessed here:

http://www.biomedcentral.com/1472-6963/11/96/prepub
